# Host–Guest Metal–Organic Frameworks-Based Long-Afterglow Luminescence Materials

**DOI:** 10.3390/molecules29132989

**Published:** 2024-06-23

**Authors:** Zhi-Chen Zhang, Zhi-Gang Gu, Jian Zhang

**Affiliations:** 1 State Key Laboratory of Structural Chemistry, Fujian Institute of Research on the Structure of Matter, Chinese Academy of Sciences, Fuzhou 350002, China; zhangzhichen@fjirsm.ac.cn (Z.-C.Z.); zhj@fjirsm.ac.cn (J.Z.); 2 College of Chemistry, Fuzhou University, Fuzhou 350108, China; 3 Fujian Science & Technology Innovation Laboratory for Optoelectronic Information of China, Fuzhou 350108, China

**Keywords:** long afterglow, MOFs, Metal-Organic framework, host-guest

## Abstract

Long-afterglow materials have a broad of applications in optoelectronic devices, sensors, medicine and other fields due to their excellent luminescent properties. The host-guest long-afterglow MOFs material combines the advantages of multi-component characteristics and the stability of MOFs, which improves its luminous performance and expands its other properties. This review introduces the classification, synthesis and application of host-guest MOFs materials with long afterglow. Due to their rigid frames and multi-channel characteristics, MOFs can load common guest materials including rare earth metals, organic dyes, carbon dots, etc. The synthesis methods of loading guest materials into MOFs include solvothermal synthesis, post-encapsulation, post-modification, etc. Those long-afterglow host-guest MOFs have a wide range of applications in the fields of sensors, information security and biological imaging.

## 1. Introduction

Photoluminescence (PL) is the luminescence of light-emitting substances that is directly excited by light [1], which can be divided into single-line exciton fluorescence and three-line exciton phosphorescence. Chromophores can absorb ultraviolet/visible light. After absorbing the energy of the incident light, the electrons in the molecular orbital transfer from the ground state to the excited singlet state with the same spin multiplicity. Then, fluorescence is generated when electrons transition between singlet states. However, the generation of phosphorescence involves intersystem crossing from excited singlet to triplet excited states, followed by a transition between the triplet excited state and the singlet ground state. Since the transition from the triplet to the ground state is spin-forbidden by quantum mechanics, it can only occur through intersystem crossing between the singlet and triplet systems. Therefore the lifetime of phosphorescence is usually longer than that of fluorescence [2].

**Figure 1:**
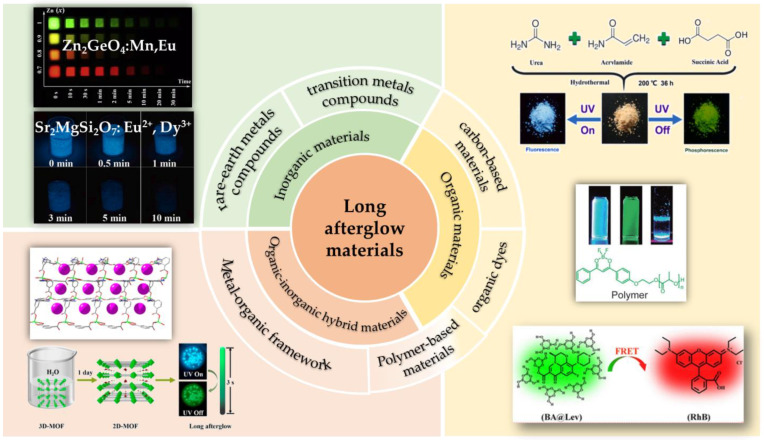
The representative examples of the classification of long-afterglow materials: inorganic materials (green); organic materials (yellow); organic-inorganic hybrid materials (orange).

Long-afterglow refers to the afterglow that continues after excitation [3], with a lifetime more than 0.1 s according to the resolving limit of the naked eye. The possible mechanisms of long afterglow include three key steps: ionization, transportation and liberation. Firstly, under photon irradiation, charge carriers (electrons and holes) are generated, which is known as ionization. Then, charge carriers can be subsequently trapped at impurity levels. When subjected to appropriate stimulations, these trapped charge carriers can gradually be released as photons, resulting in long-afterglow luminescence. Long afterglow materials, also named long lasting phosphors materials, persistent luminescence materials or energy storage materials, have attracted great attention during the past few decades due to their lasting luminescence which continues for a considerable time after the light source is switched off. The research into long-afterglow materials mainly focuses on the control of morphology, luminescent band and afterglow time [4]. Their approximate classification is shown in Figure 1. For inorganic afterglow materials, the long decay time of persistent luminescence is generally believed to be due to the fact that the excitation energy is stored in the trap and gradually released from the trap with the help of thermal energy [5]. Inorganic long-afterglow materials are composed of either transition metals compounds [6] or rare-earth metal compounds [7], mainly including rare-earth-doped aluminate [8,9], silicate [10,11,12], stannite [13], phosphate [14,15], gallate [16,17] and germanate [18,19], which usually require high-temperature calcination to obtain. Organic materials with long afterglows include carbon-based materials [20,21], organic dyes [22,23], polymer-based materials, [24,25,26,27], etc. However, it is the high cost and relatively complex synthesis that have limited the applications of those materials. Generally, the afterglow luminescence in organic systems comes from the slow radiative decay of long-lived excitons in triple excited state [28]. Since the luminescence process of long-lived excitons generally involves the transition from spin-allowed singlet states to spin-forbidden triplet states, and the triplet excitons are easy to be quenched, obtaining organic long-afterglow materials with high efficiency and long decay lifetimes has encountered significant obstacles [29].

Hybrid materials can use intermolecular interactions (such as hydrogen bonds and chemical bonds) [30] to enhance the rigidity of molecular conformation and limit molecular motion/vibration, thus reducing the non-radiation attenuation of triplet excitons, and improving their optical emission lifetime and quantum efficiency. Those hybrid materials have a wide range of applications due to its adjustability, such as gas storage [31], optical devices [32], chemical sensing [33] and catalysis [34], etc. Metal-Organic frameworks (MOFs) as a class of inorganic-organic coordination hybrid materials, have micro/nanosized structures connected with metal ions or clusters through organic ligands [35]. As organic-inorganic hybrid materials, MOFs can provide luminescence in groups, and charge transfer between metals and ligands can cause a specific luminescence [36,37]. Compared with only inorganic and organic long-afterglow materials, MOFs are emerging as room-temperature phosphorescence (RTP) materials because of their intrinsic structure features [38]; for example, high-crystalline frameworks [39], various metal cluster units [40], strong coordination interactions between metal and organic ligands [41] and the controllable formation of dense frameworks [42]. All of them help to accelerate triplet generation and produce long-afterglow emissions. However, research into afterglow of MOFs is still in its infancy [43].

PL MOFs mainly include the MOFs containing noble metals and rare-earth elements or organic luminescent materials as ligands. The luminescence principle is derived from linker-based emission, metal-based emission, antennae effects, excimer and exciplex emission, etc. [44]. Antimagnetic ions such as Zn^2+^ [45], Cu^2+^, Cd^2+^ [46] are usually selected as metal centers, light-emitting organic ligands are used as chromophores or light-emitting metal ions (such as Eu^3+^ and Tb^3+^) [47,48] are used as metal centers and organic ligands are used as sources of antenna effects, which can lead to PL MOFs [49].

**Figure 2:**
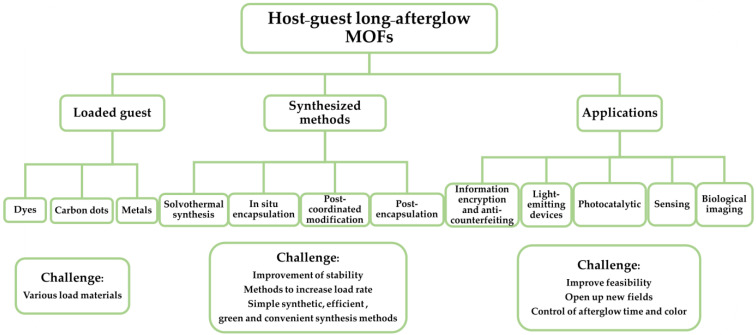
The summary of host-guest long-afterglow MOFs in loaded-guest synthesis methods and applications.

Host-guest assembly is an effective method for obtaining a long afterglow, and the rigid skeleton of MOFs makes them a promising host material. Due to the high porosity, tunable channels, stable structure and diverse composition of MOFs [50,51,52], they can be used as advantageous host materials [53,54]. Based on multi-component systems, MOF host materials can be used to regulate luminescence [51,55] and have been widely used in fields such as bio-catalysis [56], batteries [57,58] and circularly polarized optics [59]. Long afterglow can be achieved by introducing luminescent or induced luminescent guest molecules into the channels of MOFs. The metal ions in MOFs participate in the heavy ion effect, and their rigidity and confinement minimize the non-radiative loss of excitons, which can promote the generation and enhancement of afterglow. So far, plenty of review articles have explained the synthesis procedure, properties and applications of long-afterglow materials. However, there has still not been a review dedicated to host-guest MOFs-based long-afterglow materials. In this work, the progress within research into host-guest MOFs-based afterglow is reviewed, and its synthesis methods mainly include solvothermal synthesis, in situ encapsulation, post-encapsulation, post-coordinated modification, etc., and its applications in optoelectronic, anti-counterfeiting, sensing and other fields are introduced, as shown in Figure 2.

## 2. Synthesis and Classification of Afterglow Host-guest MOFs

### 2.1. Synthesis Strategy for Mono-Component Afterglow PL MOFs

Mono-component afterglow MOFs are only composed of one kind of MOF material, and their afterglow emissions also come from the luminescence groups of the metal nodes and organic linkers in the MOFs, or it may be the case that the charge transfer between the metal and the ligand in the MOFs materials can bring about a specific luminescence. However, their luminescence lifetime is short and some MOFs only exhibit phosphorescence at low temperatures (77 K), indicating that nonradiative deactivation is dominant at room temperature [60]. Common methods for synthesizing mono-component afterglow MOFs include hydro(solvo)thermal, self-assembly, etc.

**Figure 3:**
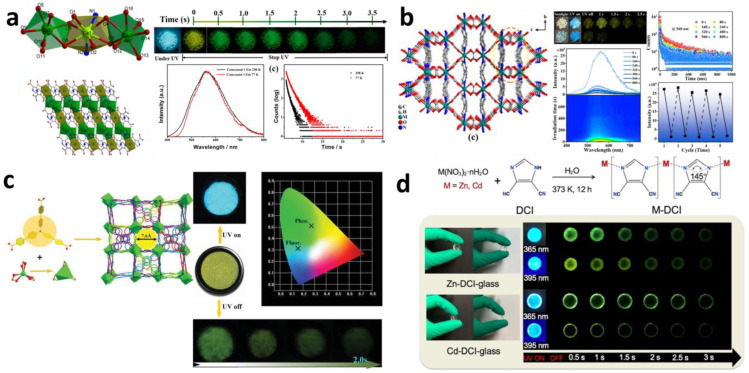
(**a**) Structureafterglow, phosphorescence emission spectrum and phosphorescence lifetime decay curves of CaMOF. Reprinted (adapted) with permission from [43]. Copyright American Chemical Society. (**b**) The 3D structure of MOF-M (M = Zn, Cd, Co, Mn), 3D structure of MOF-M (M = Zn, Cd, Co, Mn) and photos of Zn^2+^-(HCOO)_2_(4,4′-bipy) (HCOO)_2_(4,4′-bipy) before and after irradiation (λex = 365 nm), and delayed PL emission spectra of different irradiation time, long-life RTP lifetime decay curve at different irradiation time and reversible RTP intensity Reprinted (adapted) with permission from [65]. Copyright 2021Wiley-VCH GmbH. (**c**) Crystal structure, luminescence photos and CIE coordinate of fluorescence and phosphorescence emission of [(Cd^2+^)((CH_3_)_2_NH_2_)_6_][Cd_8_(TCPA)_8_]. Reprinted (adapted) with permission from [66]. Copyright 2020 Wiley-VCH GmbH. (**d**) Synthesis process of MOF glass and photos of MOF glass taken before and after turning off UV light. Reproduced with permission from ref. [67]. Copyright 2022 Wiley-VCH.

Hydro(solvo)thermal synthesis is one of the most common methods for the synthesis of mono-component afterglow PL MOFs. X. Yang et al. [45] synthesized [Zn(TPA)(DMF)](1-DMF) using the solvothermal method, with an afterglow of about 4 s. J. Liu et al. [49] prepared a unprecedented Zn-triazole MOF material, ECUT-137 by hydrothermal method, that shows a long persistent luminescence for up to 3 s. D.F. Lu et al. [43] prepared Ca-MOF [Ca_3_(IDC)_2_(DMF)_2_(HCOO)_2_] (H_2_IDC = 4,5-imidazoledicarboxylic acid; DMF = N, N-dimethylformamide). As shown in Figure 3a, its phosphorescence lifetime is about 648 ms at 298 K, and the long afterglow lasts for about 4 s. J. Liu et al. [61] synthesized micrometer-sized crystals of Cd-based MOFs Cd (m-BDC) (BIM) through the solvothermal reaction. F.Y. Cao et al. [62] prepared two mixed crystals, [Zn_4_(HEDP)_2_(TIMB)]·H_2_O and [Zn_4_(HEDP)_2_(TIMB)], by introducing 1,2,4,5-tetrakis(imidazol-1-ylmethyl)benzene (TIMB) into the zinc diphosphonate system via a solvothermal reaction, where HEDP is the 1-hydroxyethylidene diphosphonate phosphonic acid ester. [Zn_4_(HEDP)_2_(TIMB)]·H_2_O not only has fluorescence emission but also blue and green phosphorescence emission with lifetimes of 29.4 ms and 201.0 ms, respectively. [Zn_4_(HEDP)_2_(TIMB)] has a weaker phosphorescent emission. Z. Wang et al. [63] obtained a series of crystalline MOF materials through the solvothermal reaction of a CdII salt and terephthalic acid in different molar ratios and solvent mixtures at 90 °C for 48 h. These materials exhibit a long phosphorescence decay lifetime on the millisecond time scale, with an afterglow time of about 4 s after turning off the UV excitation (365 nm). J. M. Seco et al. [64] prepared four kinds of MOFs: {[Zn_2_(μ_4_-bdc)_2_(μ-pbptz)]·2DMF·3H_2_O}n, {[Cd(μ_3_-bdc)(μ-pbptz)]·3DMF}n, {[Cd_3_(μ_5_-btc)_2_(μ-pbptz)]·2DMF}n, and {[Zn_2_(μ-dhbdc)_2_(μ-pbptz)(DMF)_4_]·2DMF·H_2_O}_n_ (where bdc = ben-zene-1,4-dicarboxylato, btc = benzene-1,3,5-tricarboxylato, dhbdc = 2,5-dihydroxobenzene-1,4-dicarboxylato, pbptz = 3,6-bis(4-pyridyl)-1,2,4,5-tetrazine, DMF = N,N-dimethylformamide) through the solvothermal reaction. Under UV steady-state excitation, all the MOFs showed blue emission. {[Cd_3_(μ_5_-btc)_2_(μ-pbptz)]·2DMF}_n_ has the strongest emission, and a bluish green afterglow is still observed for about one second after the excitation source is removed.

Ma et al. [65] prepared Zn(HCOO)_2_(4,4′-bipy) that has an afterglow for 2.5 s which can be clearly recognized with the naked eye in Figure 3b. New core-shell heterogeneous MOF layered single crystals were prepared by solution-mediated epitaxy, namely Co(HCOO)_2_(4,4′-bipy)@Zn and Mn(HCOO)_2_(4,4′-bipy)@Zn. The shell has an afterglow emission, which can be used for potential space/time resolution information encryption and anti-counterfeiting applications.

Fu et al. [66] successfully synthesized a homochiral Metal-Organic framework [(Cd^2+^) ((CH_3_)_2_NH_2_)_6_] [Cd_8_(TCPA)_8_] with excellent thermal and chemical stability and porosity (22 993.2 Å_3_) via the coordination-induced assembly strategy and the use of achiral triphenylamine molecules as building blocks. [(Cd^2+^) ((CH_3_)_2_NH_2_)_6_] [Cd_8_(TCPA)_8_] shows circularly polarized luminescence and phosphorescence at room temperature, with a yellow-green afterglow that lasts for about 2 s (Figure 3c).

B. Zhou et al. [67] describe a new class of monolithic and transparent MOF bulk glasses (Zn-DCI-glass and Cd-DCI-glass) (Figure 3d), which were fabricated through solvent-assisted direct quenching, a bottom-up self-assembly strategy, and exhibited ultralong all-RTP with the PLQY up to 75% and 58.4%, respectively. Under UV excitation, both Zn-DCI-glass and Cd-DCI-glass exhibited bright blue luminescence, and a green afterglow which persisted for at least 3 s.

### 2.2. Synthesis Strategy for Host-guest MOF Afterglow Materials

Since some luminescent materials have poor stability in some practical applications and are easily destroyed in air, they can be doped into highly rigid MOFs. When guest molecules are doped, triplet state excitons are stabilized by host-guest interactions which produce afterglow emissions, and the excitation energy can be stored in host-guest materials which can continue to emit afterglow for more than a few seconds after the excitation source has been removed [68]. As a class of porous materials, the introduction of guest molecules into MOFs can also enhance channel stiffness and avoid deformation of the internal topology. In addition, the introduction of guest molecules with a long afterglow can bring unexpectedly long-lived and multi-color luminescence to MOFs [36].

The main guest materials introduced into MOFs are dye molecules, carbon dots (CDs) [69] and other complexes. In some cases, the incorporation of other chromophores into MOFs, such as dye@MOFs carbon dots@MOFs and luminescent complexes@MOFs, is an effective way to modulate the luminescence properties of MOFs [49]. We listed host-guest MOFs with long-afterglow luminescence in Table 1, and listed their phosphorescence lifetimes and colors, etc.

The methods for loading guest materials include the one-step method and the post synthesis method. The guest materials can be loaded into the MOF pores by the one-step method. However, guests are usually more likely to penetrate into the pores of MOFs in post-synthesis methods [63]. For example, if some materials are larger than the pore size, the main guest material can be synthesized by in situ encapsulation by a one-step method [70]. However, some materials are not suitable for the one-step method because MOFs are easily decomposed at high temperatures, in high acidic/basic media [70] or for other reasons. For example, CDs@MOFs are generally not suitable for one-step synthesis methods because the precursors of CDs would influence the crystallization of MOFs and the synthesis temperature of MOFs is usually lower than the carbonization temperature of CDs. The synthetic strategies such as encapsulation, pyrolysis, impregnation, and post-modification could be utilized to introduce CDs into MOFs [71]. Therefore, the main guest-loaded MOF afterglow materials can be synthesized by coordinated post-synthetic modification, post-synthetic encapsulation and other methods. The post synthesis method cannot easily damage the structures of the MOFs themselves, but improving load efficiency and simplifying the method are key factors.

#### 2.2.1. Solvothermal Synthesis

Solvothermal synthesis is the most commonly used one-step method for the synthesis of host-guest MOF materials.

Yang et al. [72] doped trace lanthanide ions into a cadmium-based afterglow CP by one pot hydrothermal synthesis. Lanthanide ion-doped coordination polymers (CPs) show an unusual red/green afterglow and a long luminescence life (Eu^3+^ is 10.54 ms, Tb^3+^ is 57.66 ms). H. R. Fu et al. [62] obtained series of [(Cd^2+^) ((CH_3_)_2_NH_2_)_6_] [Cd_8_(TCPA)_8_] · (Rhodamine B) and [(Cd^2+^) ((CH_3_)_2_NH_2_)_6_] [Cd_8_(TCPA)_8_] · (Coumarin 6) was prepared via the one-pot method. A DMF solution of Rhodamine B or Coumarin 6 was added to the synthesis system, and the dye molecules were embedded into the framework during the growth of crystals. Similarly, a series of Rhodamine B and Coumarin 6 mixed solutions with different volume ratios were added to obtain compositions of [(Cd^2+^) ((CH_3_)_2_NH_2_)_6_] [Cd_8_(TCPA)_8_] · (Rhodamine B and Coumarin 6). The series of dye-encapsulated composites showed unique fluorescence/phosphorescent double emission. Moreover, the confinement and discrete effect could efficiently diminish the aggregation-caused quenching effect of dyes and further enhance the luminescence quantum efficiency. Thus, the emission of composites can be modulated smartly via adjusting the species and contents of luminescent dyes. [(Cd^2+^) ((CH_3_)_2_NH_2_)_6_] [Cd_8_(TCPA)_8_] · (Rhodamine B and Coumarin 6) systems exhibit white-light emission.

Wu et al. [73] selected two phosphorescent ligands, isophthalic acid (IPA) and 2-methylimidazole (MIM), and synthesized a Zn (II) based-nonporous coordination polymer {Zn (IPA)(MIM)_2_}, which showed a lasting phosphorescent lifetime of 552 ms at room temperature and a strong green afterglow of 3 s after removal of the light source. The doping of eosin Y dye molecules can adjust the emission of photoluminescence to a large extent, provide long-term red phosphorescence at room temperature and expand the light capture range to the visible region.

Liu et al. [61] successfully synthesized a series of structurally stable long-wavelength and long-life luminescent MOFs by encapsulating different dyes into the green phosphorescent MOFs such as Cd(m-BDC) (BIM) via a solvothermal approach. Cd (m-BDC) (BIM) (MOF PM 1) phosphorescence lasts for about 10 s. Cd (m-BDC) (BIM) 3–6 exhibit MOF-dye dual long-lived luminescence emissions that last for a few seconds as traced by the naked eye and show yellowish green, yellow, orange, and red delayed fluorescence from the encapsulated dyes.

**Figure 4:**
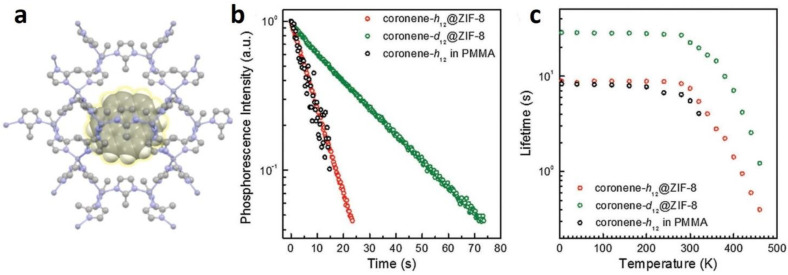
(**a**) Structure of coronene@ZIF-8. (**b**) Phosphorescence decay profiles and (**c**) temperature dependence of τ_phos_ of coronene-h_12_ doped into PMMA, coronene-h_12_@ZIF-8, and coronene-d_12_@ZIF-8 (green circles) at room temperature. Reprinted (adapted) with permission from [60]. Copyright 2016 Wiley-VCH.

Mieno et al. [60] incorporated coronene-h_12_ and coronene-d_12_ emitters into the cavities of ZIF-8 during the synthesis of the framework according to the fabrication method for fluorescein-encapsulated ZIF-8 (Figure 4). Polyhedral crystals of coronene-encapsulated ZIF-8 (hereafter referred to coronene-h_12_@ZIF-8 and coronene-d_12_@ZIF-8) were obtained. The phosphorescence lifetime of coro-nene-h_12_@ZIF-8 at 300 K (7.4 s) was similar to that at 5 K (8.8 s), and the phosphorescence lifetime of coronene-d_12_@ZIF-8 at 300 K was τ_phos_ (22.4 s). Yang and Yan [74] prepared an anionic MOF, [CdLi(IPA)_2_](Me_2_NH_2_) (IPA = isochthalic acid, Me_2_NH_2_ = dimethylamine), using the solvothermal method. The single crystal structure analysis shows that IPA electron rich benzene ring is arranged in the [CdLi(IPA)_2_](Me_2_NH_2_) channel and occupied by an exchangeable (Me_2_NH_2_)^+^ guest cation. The desired Mn^2+^-doped [CdLi(IPA)_2_](Me_2_NH_2_) material with a tunable green to red long-lasting phosphorescence emission was realized by ion-exchange with Mn^2+^ ions at different concentrations. and the phosphorescent lifetime of Mn^2+^@ [CdLi(IPA)_2_](Me_2_NH_2_) is about 32 ms.

Liu et al. [75] designed a new class of Cd-MOFs using 4,5-imidazole dicarboxylic acid (IMDC) as the ligand at 140 °C for 72 h. The experimental results showed that the incorporation of N, N-dimethylformamide (DMF) into MOF (Cd-DMF) exhibited a dual-phosphorescence emission, which occurred through a combination of ligand-to-ligand charge transfer (ca.560 nm, τ = 187.0 ms) and ligand self-emission (ca. 530 nm, τ = 196.0 ms) due to structural distortion. In addition, upon excitation by a commercial white LED light source, a bright orange-yellow afterglow was observed for about 4 s.

**Figure 5:**
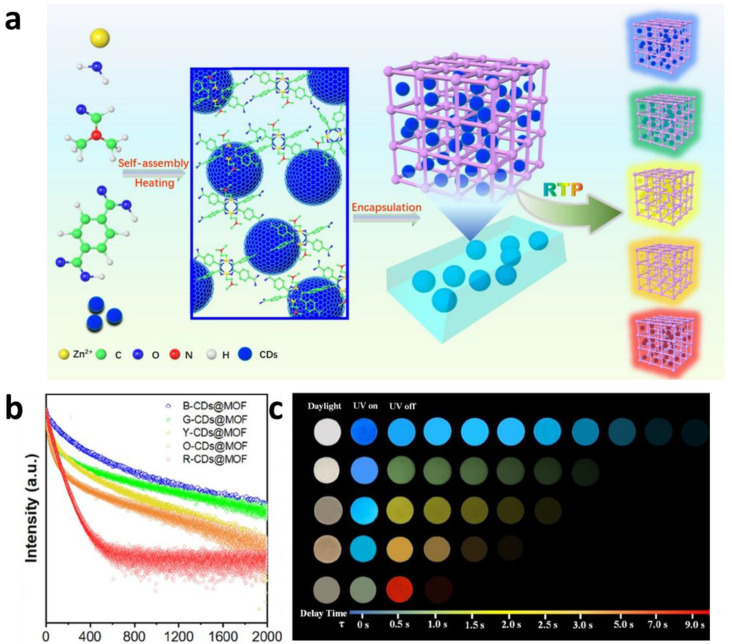
(**a**) Schematic illustration of the hydrothermal procedure for preparing CDs@MOF composites with room-temperature RTP emissions. (**b**) Time-resolved RTP spectra. (**c**) Photographs of the five CDs@MOF composites captured in daylight and with a UV lamp (365 nm) ON or OFF. Reprinted (adapted) with permission from [76]. Copyright 2022 American Chemical Society.

Xu et al. [76] used one-step hydrothermal synthesis CDs@MOF. The synthesis of parent fluorescent CDs includes mixing appropriate carbon source and heteroatom dopant in aqueous solution, and then heat treating in water at 200 °C for 6 h. Then, synthesize by directly dispersing fluorescent CDs into an MOF growth solution to create a CDs@MOF composite material, as shown in Figure 5. The developed CDs@MOF composites exhibited RTP with a tunable emission wavelength from blue (478 nm) to red (631 nm), while the lifetime was tunable from 85.67 to 1064.21 ms. The CDs@MOF composites emitted bright blue or green luminescence upon UV (365 nm) illumination and then emitted blue, green, yellow, orange, and red RTP emissions after the 365 nm UV illumination ceased. The RTP emissions persisted for ~1.0 to 9.0 s under ambient conditions.

Liu et al. [77] exhibited thermally activated delayed fluorescence (TADF) that restricts electron-rich triphenyl (Tpl) donors to cage-shaped MOF hosts (NKU-111) with electron-deficient 2,4,6-tris(pyridin-4-yl)-1,3,5-triazine (Tpt) receptors as ligands through solvent thermal reactions. The delayed PL spectrum at 77 K shows that the maximum phosphorescence emitted by the lowest excited state T1 is around 515 nm, with a lifetime of 3.23 s. At 288 K, the fluorescence emission time reaches 0.18 s near 492 nm.

#### 2.2.2. In Situ Encapsulation

In situ encapsulation is a simple one-step synthesis method.

Z. Zhao et al. [78] designed and assembled an intelligent nanoprobe, Gd[PC]@ZIF-8, by homogenously encapsulating a rare-earth complex phosphor Gd[(Pyr)4cyclen] (Pyr = pyrenol) into a zeolitic imidazolate framework (ZIF-8) through one-pot synthesis. Compared with the pure complex, the nanoprobe Gd[PC]@ZIF-8 exhibited excellent RTP properties, including a strongly enhanced phosphorescence intensity and quantum yield and a highly elongated decay lifetime, due to the limitations of the MOF. Knedel et al. [79] obtained ([PtCN(L)]@ZIF-8 and [PtCl(L)]@ZIF-8) by the in situ encapsulation of the Pt (II) complex in ZIF-8. For [PtCN(L)]@ZIF-8-2, the lower loading leads to an enhanced lifetime (τ = 18.3 μs) and to a boost in ΦL reaching 0.29 in an Ar atmosphere, if compared with the solid state of the pure complex.

Zinc isophthalic acid (IPA)-based MOF microcrystals can be facilely synthesized in aqueous solution with a long-lasting lifetime of 900 ms. The in-situ encapsulation of rhodamine B into the MOF matrix can expressly tune the phosphorescence emission color from green to red, enhancing the ability of solar energy harvesting, charge carrier separation efficiency and optoelectronics performance. Zn (IPA) MOF has green phosphorescent emissions, the main peak is 487 nm and the lasting phosphorescent lifetime is 926.56 ms. After removing the ultraviolet light, the green emissions can be easily recognized by the naked eye, and gradually fade with a few seconds of significant persistent light. The Rhodamine B @Zn(IPA)-MOF obtained by in situ encapsulation of Rhodamine B into a MOF matrix shows a rare example of a red RTP with an effective PET-based lifetime of 450 ms [80].

#### 2.2.3. Post-Coordinated Modification

**Figure 6:**
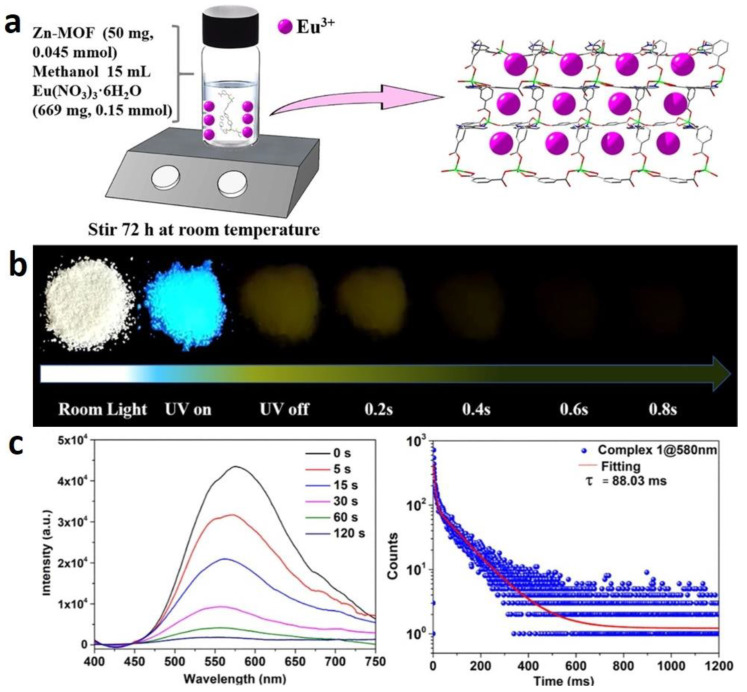
(**a**) Synthesis diagram of Eu^3+^@Zn-MOF. (**b**) Photographs of Eu^3+^@Zn-MOF before and after irradiation by UV light. (**c**) Time-dependent luminescence (phosphorescence) spectra of Eu^3+^@Zn-MOF before and after irradiation (λ_ex_ = 370 nm) and long-lived RTP lifetime decay curve at 580 nm. Reprinted (adapted) with permission from [78]. Copyright 2022 American Chemical Society.

The Eu-supplemented version of Zn-MOF was obtained by coordinated post-synthetic modification. Eu^3+^@{[Zn_2_(cbbpy)_2_(IPA)_2_]·4H_2_O}_n_ shows intense pink emission under 365 nm UV. {[Zn_2_(cbbpy)_2_(IPA)_2_] · 4H_2_O}_n_ displays blue luminescence in Figure 6. After ceasing the UV (365 nm) irradiation, an afterglow with yellow persistent luminescence can be recognized by the naked eye for 0.8 s. In addition, upon 395 nm excitation, Eu^3+^@{[Zn_2_(cbbpy)_2_(IPA)_2_]·4H_2_O}_n_ shows white light emissions, which implies that the incorporation of a Eu^3+^ ion and isophthalic acid facilitates the modulation of luminescence [78].

#### 2.2.4. Post-Encapsulation

The synthesis of MOF-5 was carried out in DMF, but since complexes are poorly soluble in this solvent, the in situ approach was not tried for MOF-5. MOF-5 composites ([PtCN(L)]@MOF-5, [PtCl(L)]@MOF-5) were obtained by post-synthetic encapsulation. For the [PtCl(L)] @MOF-5 composite as a powder, the ΦL is actually lower (≤0.04) than in the solid state (ΦL = 0.04). Furthermore, the τ values of MOF-5 composites obtained from powders (11.1 and 10.5 μs) are longer than that of single-crystal MOF-5 (4.7 and 4.04 μs) [79]. By introducing effective noncovalent interactions and enhancing π-π stacking interactions, metal cages composed of a metal center and an organic ligand with effective host-guest chemistry can be constructed. Hou et al. [81] used the method of multi-component self-assembly to obtain a barrel-shaped metal cage with high emissions. In addition, these two metal cages can effectively encapsulate polycyclic aromatic hydrocarbons (pyrene, triphenylyne and perylene), providing a way to fine tune the host-guest system emission. Due to the complementary emission of the metal cage and perylene, white light emission is obtained. Yang et al. [82] prepared a caged-coronene system (CC) through post self-assembly modification. The synthetic encapsulation procedure induced core-to-cage charge transfer. The package-induced core-to-cage charge transfer (CCCT) enhances the absorption of visible light and extends the tri state lifetime.

He et al. [83] functionalized the surfaces of PLNPs (Zn_3_Ga_2_Ge_2_O_10_/0.5% Mn), then stirred them with NH_2_-MIL-101 (Fe) in an aqueous solution to obtain PLNPs/MOF composite materials. This material is formed by covalent bonding between Zn_3_Ga_2_Ge_2_O_10_/Mn and NH_2_-MIL101 (Fe), with an afterglow time of over 16 days. It is used for the photodegradation of RhB and the photoreduction of Cr (VI).

#### 2.2.5. Other Synthesis Methods

Other synthesis methods include the anion exchange method, introducing guest molecules into MOFs. Reversible guest molecular loading can be achieved by solvent immersion loading and heating and removal.

Fu et al. [66] prepared [(Cd^2+^)((CH_3_)2NH_2_)_6_] [Cd_8_(TCPA)_8_]·(Acriflavine) 1–8 composites via the anion exchange process. They soaked [(Cd^2+^) ((CH_3_)_2_NH_2_)_6_] [Cd_8_(TCPA)_8_] in an DMF solution of acridine (5 × 10^−3^ mol L^−1^), until the anion exchange process reached dynamic equilibrium when the composite was obtained.

**Figure 7:**
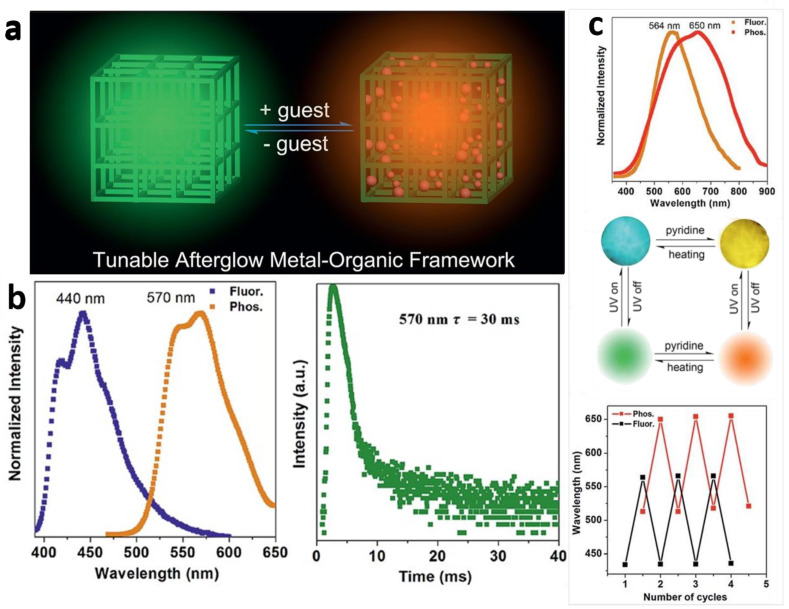
(**a**) Schematic representation of the tunable afterglow MOFs based on host-guest interactions. (**b**) Normalized photoluminescence spectra and time-resolved phosphorescence decay curves of 1- Pyridine. (**c**) Normalized photoluminescence spectra for MOF-5 after treatment with Pyridine, fluorescence and phosphorescence of MOF-5 crystals before and after pyridine treatment and the reversible change of wavelength. Reprinted (adapted) with permission from [45]. Copyright 2016 Royal Society of Chemistry.

Yang et al. [45] report that two types of Zn-terephthalate (TPA) MOFs (namely [Zn(TPA)(DMF)] (1-DMF) and MOF-5) could exhibit an obvious room-temperature afterglow emission with a time-resolved luminescence lifetime as high as 0.47 s. In addition, soaking MOF-5 crystals with Pyridine solvent enables the introduction of guest molecules into the MOF nanochannels, and the obtained Pyridine@MOF-5 samples can exhibit highly tunable phosphorescent colors (from cyan to yellow and from green to red, respectively). Further heating of the Pyridine@MOF-5 samples was able to remove the Pyridine between the channels (Figure 7).

Ni et al. [84] have reported a new doping strategy to prepare RTP composites that can be achieved by water-stimulated matrix structural transformation. In the process of the solvothermal synthesis of MOF-5, phosphors were loaded into the channel of MOF to prepare a series of water-awakened RTP phosphor@MOF composites with an RTP color ranging from green to orange-red and durations of 3–6 s. Research shows that water can lead to the conversion of MOF-5 into a more compact structure and that the water molecules in the channel form a strong hydrogen bond network, which limits the movement of the ligand and stabilizes the triplet state, leading to RTP.

**Table 1:** The organic ligands and encapsulated guest molecules in long-afterglow MOF materials.

Code	Ligand	Coordinated Metal Ions	Guest	Phosphorescence Lifetime (ms)	Afterglow Color	T (K)	Ref.
1	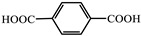	Zn^2+^	Pyridine	470	Green	RT	[45]
2	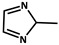	Zn^2+^	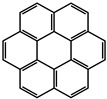	7400 22,400	Green	300	[60]
3	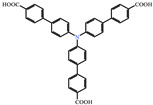	Cd^2+^	Coumarin 6, Acriflavine, Rhodamine B	1180–672	Green, yellow, and red in DMF solution	RT	[66]
4	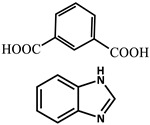	Cd^2+^	4-Methylumbelliferone, Fluorescent Green B, Rhodamine 123, Rhodamine 6G, Rhodamine B	293–765	Green, yellow, orange, to red.	RT	[61]
5	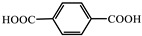	Cd^2+^	Eu^3+^ Tb^3+^	10.54 57.66	Red	RT	[72]
6	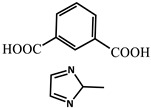	Zn^2+^	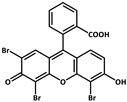	0.11, 3.57, 1.89	Green to red	RT	[73]
7	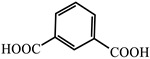	Cd^2+^, Li^+^	(Me_2_NH_2_)^+^ Mn^2+^	32 1.6–10.5	Green Green to red		[74]
8	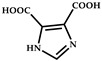	Cd^2+^	N,N-dimethylformamide	187 196	yellow to green	RT	[75]
9	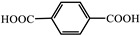	Zn^2+^	CDs	85.67–1064.21	Green	RT	[76]
10	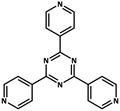	Cd^2+^	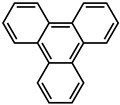	3230	Green	77	[77]
11	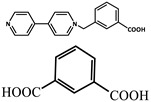	Zn^2+^	Eu^3+^	800	Yellow		[78]
12	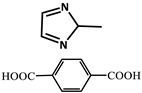	Zn^2+^	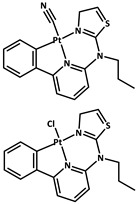	0.0184 0.0105 0.0111	Green	77	[79]
13	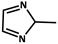	Zn^2+^	Gd[(Pyr)_4_cyclen] (Pyr = pyrenol)	0.03695	Green	77	[85]
14	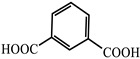	Zn^2+^	Rhodamine B	926.56	Red	RT	[80]
15	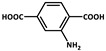	Fe^3+^	Zn_3_Ga_2_Ge_2_O_10_/Mn	280,270	Green	RT	[83]
16	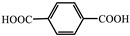	Zn^2+^	phosphors	24.52–756.63	Green to orange-red	RT	[84]

## 3. Application of Afterglow MOFs

Long-afterglow MOF materials have broad application prospects in sensing [83,86], displays [87], information security [88,89], biomedicine [90], photocatalysis [91] and other fields. Due to the advantages of host-guest afterglow MOF materials, such as the luminescent properties of the guest material and the porous channels of the host MOF material, the host-guest combination leads to longer or multi-color luminescence, making it highly advantageous for applications. Here, we present some application examples.

### 3.1. Information Encryption and Anti-Counterfeiting

Taking advantage of the multi-color luminescence and adjustable luminescence characteristics of the host-guest long-afterglow MOFs, the long-afterglow MOFs are made into an anti-counterfeiting ink, which has important applications in the information encryption and anti-counterfeiting fields.

**Figure 8:**
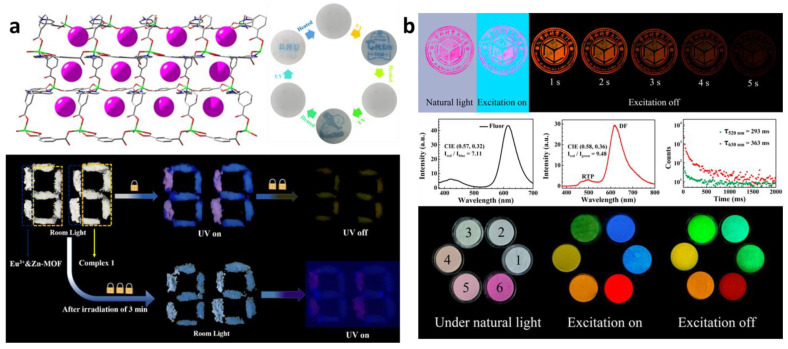
(**a**) Photographs of the inkless print and re-writability based on the photochromism of Zn-MOF and anticounterfeiting platform using persistent luminescence and photochromism of Zn-MOF and Eu^3+^@Zn-MOF. Reprinted (adapted) with permission from [78]. Copyright 2022 American Chemical Society. (**b**) Photographs of PM 1−6 and anticounterfeiting stamp. Reprinted (adapted) with permission from [61]. Copyright 2018 American Chemical Society.

Yang et al. [78] used Zn-MOF and Eu^3+^@Zn-MOF for ink-free and erasable printing and explored its application in information encryption and anti-counterfeiting. As shown in Figure 8a, the figure “88” shaped pattern is composed of Zn MOF (complex 1) and Eu^3+^@ Zn-MOF. Under the room light, No. 88 is white. Under 365 nm UV excitation, Eu^3+^@Zn-MOF shows a pink fluorescence and Zn-MOF shows blue luminescence, respectively. After the UV light is suddenly removed, only Zn-MOF shows a yellow afterglow. When the irradiation time is prolonged for 3 min, the pink number “11” appears under ultraviolet light, while the number “88” changes from white to blue under indoor light. Therefore, the real number is successfully triple encrypted by the UV exposure duration and the conditions of observation. Compared to single fluorescent anti-counterfeiting measures, long-lived luminescence can provide more anti-counterfeiting information, such as rare spectra and lifetime features. Encapsulated MOF Cd(m-BDC) (BIM) 6 anti-counterfeiting ink plates with at least two excitation and color emission (MOFs themselves and dyes, respectively) achieve an upgraded level of anti-counterfeiting measures, and this long-life luminescence anti-counterfeiting material is more difficult to be replaced by other materials. The dye-encapsulated MOFs were applied as anticounterfeiting stamps with multiple spectral features that are superior to others based on a single fluorescence in Figure 8b [61].

Wang et al. [92] reported an intelligent responsive MOF that achieves visual long-duration luminescence switching and multi photoluminescence modulation in dynamic MOFs through the absorption and elimination of water molecules, providing new ideas and perspectives for anti-counterfeiting and other applications.

Yu et al. [93] synthesized a series of MOFs with tunable luminescence, using lanthanide nitrates and 4-(2,5-dicarboxyphenoxy) phthalic acid, and mixed them with the biopolymer carrageenan to obtain film materials. These materials exhibit a blue afterglow, for up to 0.8 s, and can be directly used for anti-counterfeiting or for fabric anti-counterfeiting. Shi et al. [88] prepared two Ca-MOFs with visible afterglow durations of about 13 s and applied them within dynamic anti-counterfeiting. Wang et al. [94] prepared three metal halide complexes by blending crown ether ligands in an appropriate proportion to metal salts, exhibiting excitation-dependent room temperature phosphorescence ranging from blue/cyan to green/yellow. This material shows enormous potential for applications in information storage, encryption, anti-counterfeiting and other areas.

Zheng et al. [95] adjusted the afterglow time through the use of auxiliary ligand adjustment strategies, and obtained various chiral MOF-based long-afterglow materials using photocurable 3D printing methods for anti-counterfeiting and information encryption applications.

### 3.2. Photocatalysis

The energy storage characteristics of long-afterglow materials enable them to be used as photocatalytic materials, extending the photocatalytic time without light sources **[96]**.

Chen et al. [96] prepared long-afterglow/graphitic carbon nitride@Metal-Organic framework ((SrAl_2_O_4_: Eu^2+^, Dy^3+^/g-C_3_N_4_@NH_2_-UiO-66, SGN) using a thermal solution method. After adding NH_2_-UiO-66, the degradation rates of binary materials GN and SN were significantly increased to around 60% and 80%, respectively, within 30 min. After 30 min of photocatalysis, 95% of the Methylene blue was degraded, indicating that SGN has an enhanced photocatalytic performance.

**Figure 9:**
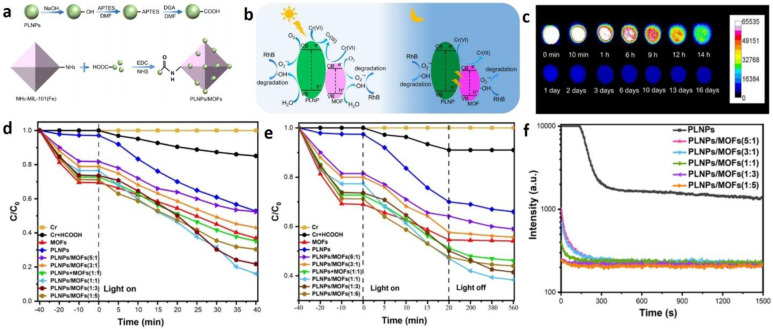
(**a**) Schematic illustration of the preparation of PLNPs/MOFs. (**b**) The mechanism of the composite photocatalyst PLNPs/MOFs for photocatalytic degradation of pollutants. (**c**) Afterglow decay images of PLNPs were recorded by a CCD camera at different times. (**d**) Photocatalytic reduction efficiency and (**e**) round-the-clock reduction efficiency of Cr (VI). (**f**) Afterglow decay curves of PLNPs and PLNPs/MOFs composites. Reprinted (adapted) with permission from [83]. Copyright 2023 American Chemical Society.

Due to the large number of active transition metal sites, large specific surface area, and visible light response, the combination of PLNPs/ MOFs provides a strategy for the development of efficient all-weather photocatalysts, with great potential in environmental protection and the utilization of solar energy. Due to the formation of heterostructures and the increase in specific surface area and active catalytic sites, PLNPs/ MOFs exhibit a high catalytic efficiency and high photocatalytic activity in the dark for 12 h (Figure 9). The photocatalytic performance is significantly higher than that of physical mixtures of PLNPs^+^ MOFs, pure PLNPs and MOFs **[82]**.

### 3.3. Sensing

Because the reduction in a material’s long-afterglow luminescence life is related to oxygen and other factors, long-afterglow MOF materials can be used for corresponding sensors.

**Figure 10:**
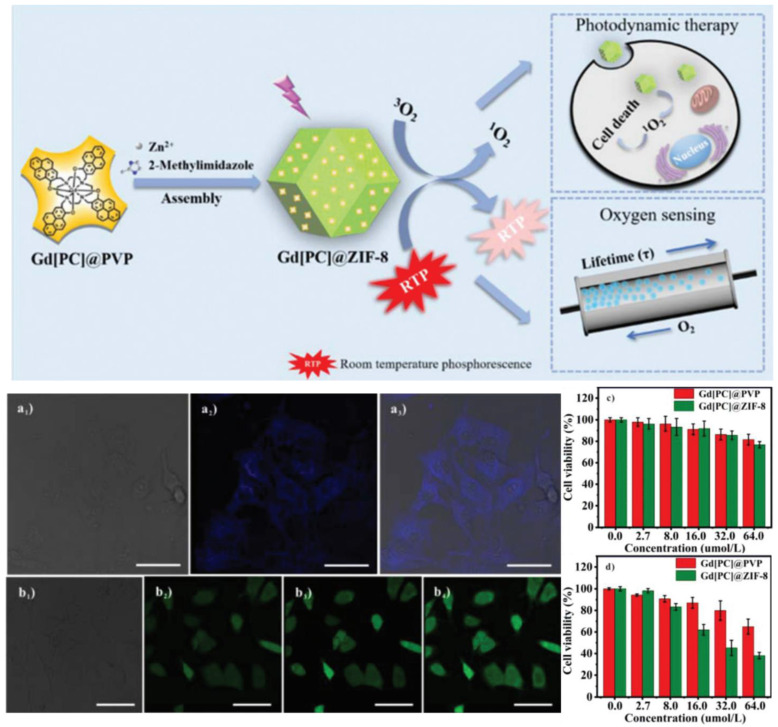
Illustration of the fabrication process of the nanoprobe Gd[PC]@ZIF-8 and its synchronous applications in PDT and oxygen sensing: (**a_1_**–**a_3_**) confocal microscopic images of HeLa cells incubated with Gd[PC]@ZIF-8@PLL for 6 h. (**a_1_**) Bright, (**a_2_**) fluorescence, (**a_3_**) merged. (**b_1_**–**b_4_**) Confocal images of intracellular ROS generation in HeLa cells treated with Gd[PC]@ZIF-8 after irradiation by a 365 nm LED lamp at different times. The cells were irradiated for 30 s every time at intervals of 5 min. (**b_1_**) Bright, (**b_2_**) 30 s, (**b_3_**) 60 s, (**b_4_**) 90 s. Scale bar = 40 μm. MTT assays for HeLa cells incubated with different concentrations of Gd[PC]@PVP and Gd[PC]@ZIF-8 (**c**) without and (**d**) with 365 nm LED lamp irradiation for 15 min. Reprinted (adapted) with permission from [85]. Copyright 2019 Royal Society of Chemistry.

Gd[PC]@ZIF-8 showed an outstanding linear relationship between the phosphorescence decay lifetime, intensity and oxygen concentration, which can be used for oxygen sensing. In addition, as a favorable photosensitizer, the Gd[(Pyr)_4_cyclen] molecules were delivered into cancer cells by the ZIF-8 nanocarrier. More oxygen molecules were converted into ^1^O_2_ under irradiation, which induced a good photodynamic therapy (PDT) efficiency against cancer cells (Figure 10) [85].

The Tb-based MOFs obtained by Yu et al. also can be used for sensing due to their high selectivity for luminescence quenching of ofloxacin, demonstrating an ultra-low detection limit (1.1 nM) and an extremely short response time. The sensing was not interfered with by the typical components of blood plasma and urine, indicating that it may be a promising luminescent sensor for real-life applications in the medical and agricultural fields [93].

Wang et al. [97] synthesized two amino-functional MOFs (VB4-2D and VB4-1D) with adjustable fluorescence and phosphorescence. The basic amino functional groups in the MOFs exhibited acid and ammonia sensitivity, which provides a possibility for use in sensors. Zhu et al. [29] proposed A novel Metal-Organic framework (MOF) with a long persistent luminescence for multilevel oxygen detection. Lv et al. [98] prepared materials (namely ZGGO@ZIF-8) using chromium-doped zinc germanate (ZGGO) near-infrared (NIR) persistent luminescent nanoparticles (PLNPs) and zeolite imidazole salt framework 8 (ZIF-8). The loading capacity of doxorubicin (DOX) is very high (LC = 93.2%), and drugs loaded with DOX are accelerated in acidic microenvironments (such as tumor cells), which may provide new opportunities for the development of advanced multifunctional nanoplatforms for tumor treatment, chemical sensing and optical information storage.

### 3.4. Light-Emitting Devices

The luminescent characteristics of long-afterglow MOFs make them have important applications in light-emitting devices. Zhou et al. [67] have integrated the Zn-DCI glass with blue luminescence and green afterglow, yellow phosphor (Ba, Sr)_2_SiO_4_:Eu^2+^ and red phosphor (Ca, Sr)AlSiN_3_:Eu^2+^ on a commercial 400 nm ultraviolet chip to fabricate white light-emitting diode (WLED) devices. The WLED shows s good color chromaticity stability driven at different low currents of 0.02 to 0.12 mA, and a high device stability after 96 h of continuous use. This work creates opportunities for applying these long-afterglow photonic glasses to information storage and light-emitting devices.

Liu et al. [75] doped Eosin Y disodium salt (EY2) and Rhodamine B (RhB) dye into Cd DMF, which is expected to be used for the white light illumination of LEDs. Wang et al. [94] prepared two MOFs based on the multi dentate ligand 2-hydroxynicotinic acid with multiple nuclear clusters, which exhibit white light and long-lasting luminescence, and have potential applications in lighting and safety systems.

## 4. Conclusions

Fluorescent materials have shown great potential in optoelectronics, imaging, sensing and secure printing. Because a long afterglow can store light energy and continue to emit light after the excitation light is turned off, long-afterglow luminescent materials, especially MOF materials, have become a research hotspot. In this review, the long-afterglow materials included organic, inorganic and organic-inorganic hybrid materials. The host-guest system has been constructed to stabilize the triplet exciton, prolong the afterglow emission life and form multi-color luminescence. MOFs are commonly used hybrid materials, which can provide rigid frames and channels for the host-guest system, so they can be used as the host in host-guest material to load guests.

We summarized the preparation methods, classification and application of host-guest MOFs, MOF-loaded dye molecules, carbon dots, rare-earth metals and other guests. Their preparation methods include solvothermal, in situ encapsulation, post-coordinated modification and post-encapsulation. These host-guest MOFs have important applications in sensors, biological imaging, information anti-counterfeiting, etc., based on their multi-color and long-term luminous properties. However, due to the easy quenching of afterglow and the limited availability of host-guest long-afterglow MOF material, research on host-guest long-afterglow MOF materials is still limited. Further research on the luminescence mechanism of host-guest long-afterglow MOF materials will facilitate the adjustment of afterglow duration and color. The preparation of host-guest long-afterglow MOF materials will develop in a more convenient, efficient, green, and inexpensive direction. The efficiency of loading the guest onto MOFs should be continuously improved to optimize performance. Moreover, the application fields of long-afterglow MOF materials should be expanded to improve their performance.

Due to the multi-color and extended afterglow time of the host-guest MOF materials, they are expected to have potential applications in film, optical devices and other fields. Research advances have been made in the application of MOF materials in films. Using MOFs to build host-guest materials to obtain a long afterglow can lead to the creation of new film materials with recognition, anti-counterfeiting and other functions. The research on host-guest MOF materials will move towards more green and convenient synthesis methods, afterglow color adjustment and practical applications of materials, etc.
